# Granulomatous Colitis Due to Hermansky-Pudlak Syndrome

**DOI:** 10.14309/crj.0000000000001477

**Published:** 2024-10-31

**Authors:** Hajar Koulali, Samira Azzmouri, Mariam Tajir, Khawla Zerrouki, Anass Haloui, Ouiam Elmqaddem, Abdelkrim Zazour, Zahi Ismaili, Ghizlane Kharrasse

**Affiliations:** 1 Department of Hepato-Gastroenterology, Mohammed VI University Hospital, Oujda, Morocco; 2 Digestive Diseases Research Laboratory (DSRL), Faculty of Medicine and Pharmacy, Mohammed First University, Oujda, Morocco; 3 Laboratoire de Génétique Médicale, Laboratoire Central, Centre Hospitalo-Universitaire Mohammed VI, Faculté de Médecine et de Pharmacie d’Oujda, Université Mohammed Premier, Oujda, Morocco; 4 Department of Pathology, Mohammed VI University Hospital/Faculty of Medicine, Mohammed 1st University, Oujda, Morocco

**Keywords:** granulomatous, colitis, Hermansky-Pudlak syndrome

## Abstract

Hermansky-Pudlak syndrome (HPS) is a rare genetic disorder characterized by oculocutaneous albinism, bleeding diathesis, and multiorgan involvement. Granulomatous enterocolitis may occur in a subset of patients. Distinguishing HPS from other diseases such as Crohn's disease can be challenging, and managing HPS-associated colitis is complex. Recent reports suggest potential efficacy of infliximab in treating HPS-related granulomatous colitis. Here, we document the case of a 27-year-old patient with genetically confirmed HPS type 1, presenting with granulomatous colitis and successfully treated with corticosteroids and infliximab.

## INTRODUCTION

Hermansky-Pudlak syndrome (HPS) is a rare autosomal recessive disorder characterized by multiorgan involvement. It is associated with oculocutaneous albinism and bleeding diathesis, which are specific to this syndrome.^1^ Other clinical manifestations vary depending on the type of HPS.^2^

Currently, 11 types of HPS have been identified, and each type is associated with distinct mutations.^2^ These mutations lead to abnormalities in the biogenesis of lysosomes and lysosome-related organelles as well as impaired intracellular protein transport.^1^

Approximately 20% of patients with HPS present with granulomatous enterocolitis; this association is predominantly observed in patients with type 1 and type 4 HPS.^1,2^ Cases of perianal involvement have also been reported in patients with HPS.^3^ Diagnosing granulomatous colitis associated with HPS can be challenging, and its management also poses significant difficulties. The case of a 27-year-old patient with granulomatous colitis associated with HPS is presented in this study.

## CASE REPORT

**Figure 1.**
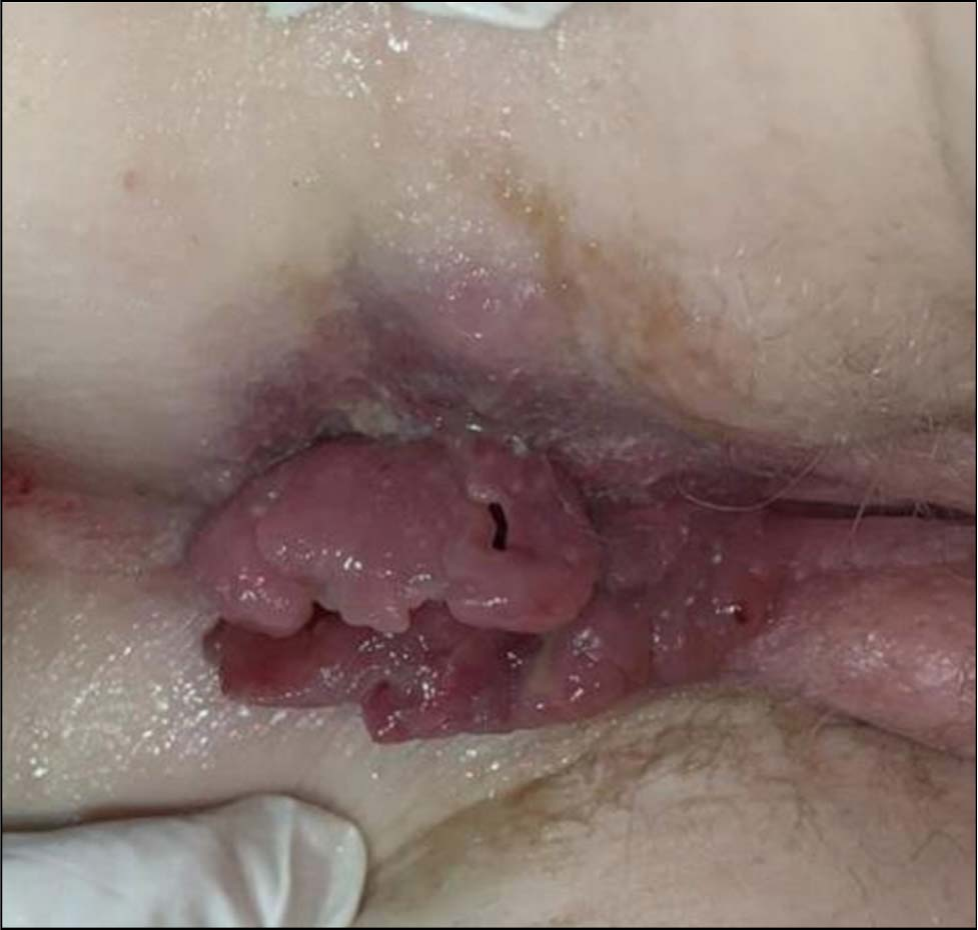
Edematous and hypertrophic skin tags.

**Figure 2.**
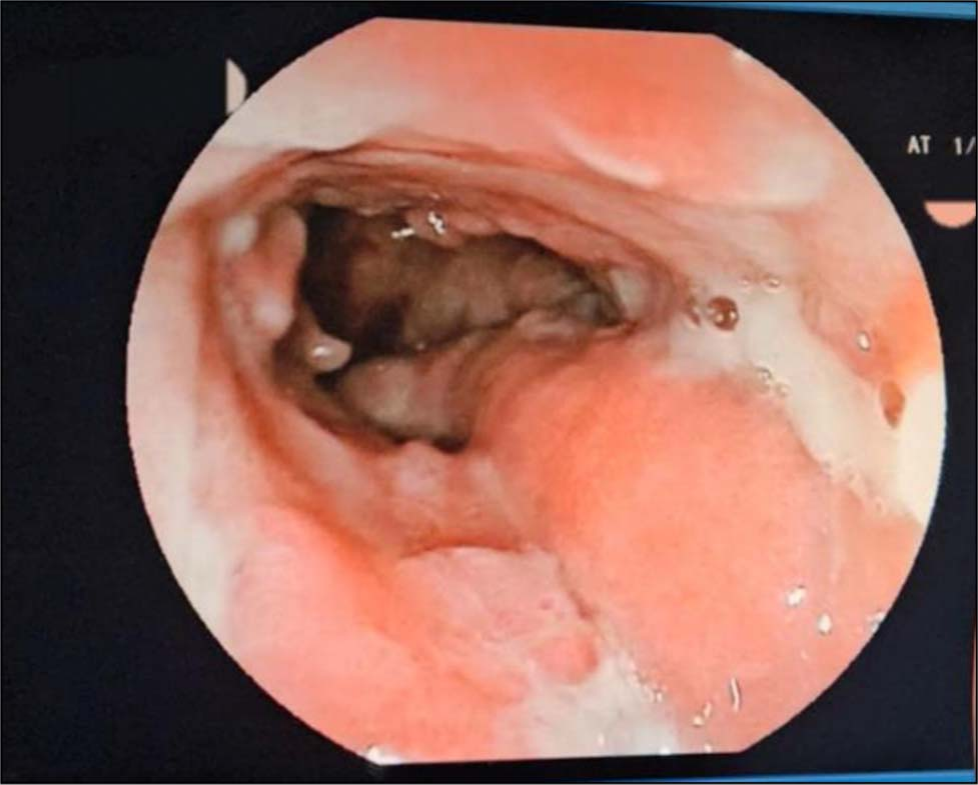
Sigmoidoscopy showing edema, erythema, and ulcerations.

**Figure 3.**
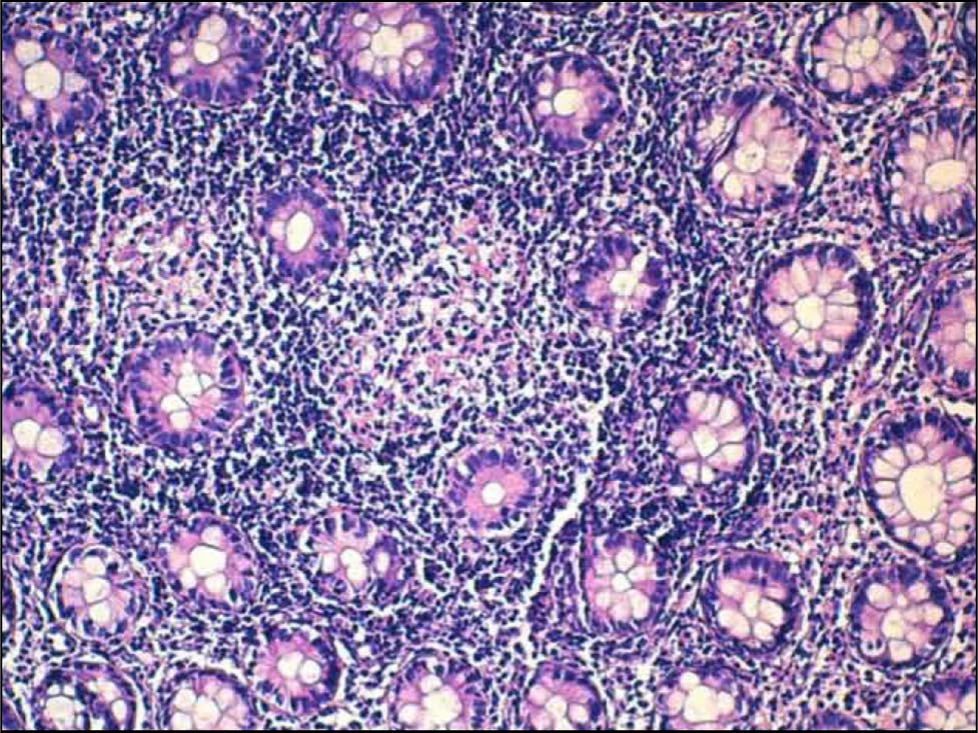
High-power view of colonic mucosa from the patient. The chorion is the site of a granuloma consisting mainly of epithelioid cells (×40).

**Figure 4.**
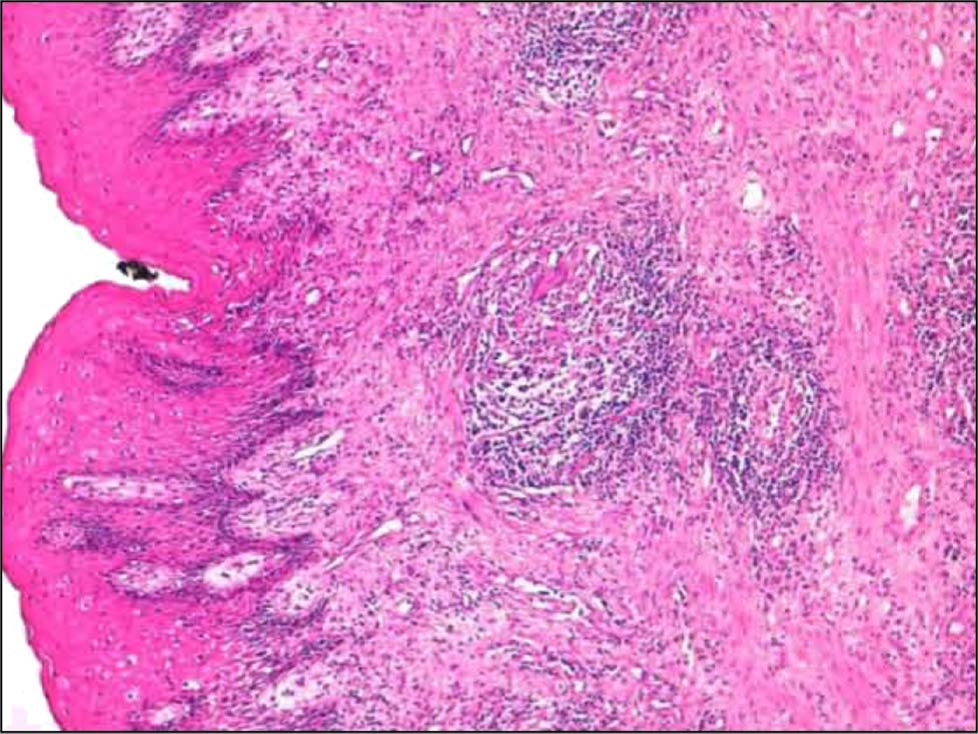
Low-power view showing the anal mucosa. The mucosa is covered by normal-looking squamous epithelium, with a moderate amount of inflammatory cells colonizing the lamina propria, as well as numerous granulomas consisting of epithelioid cells surrounded by a ring of lymphocytes and multiple giant cells (×4).

A 27-year-old woman with a history of massive post amygdalectomy hemorrhage and a family history of albinism in 2 siblings presented to the emergency department with bloody diarrhea, abdominal pain, and rectal bleeding, similar symptoms occurred over the past year, alongside weight loss and loss of appetite. The patient was severely dehydrated and malnourished; she exhibited paleness, oculocutaneous albinism, and horizontal nystagmus. Abdominal palpation revealed diffuse tenderness, while perianal examination showed swollen and ulcerated skin tags (Figure 1). Laboratory findings were as follows: hemoglobin of 11 g/dL, C-reactive protein of 118 mg/L, and hypoalbuminemia of 2.5 g/dL. Stool sample cultures and tuberculosis blood tests were negative. A computed tomography scan detected an inflammatory thickening of the rectum and sigmoid colon wall, as well as enlarged lymph nodes. Pelvic magnetic resonance imaging revealed the presence of complex anal fistulas accompanied by a small parietal abscess measuring 13 mm. A colonoscopy showed erythema, edema, and superficial ulcerations limited to the rectum and sigmoid colon (Figure 2). Histopathological examination identified multiple granulomas without caseous necrosis (Figure 3), as well as features of active colitis (including edema and crypt abscess) and chronicity (including crypt architectural distortion and transmural inflammation). The polymerase chain reaction test to detect tuberculosis in the biopsy samples was negative. Molecular genetic analysis confirmed that exon 20 of the HPS1 gene was homozygous recessive. Based on the clinical findings and the high prevalence of tuberculosis in Morocco, intestinal and perianal tuberculosis could not be ruled out. The patient was started on a 6-month antituberculosis regimen. However, she failed to respond; then, she received azathioprine without success. Corticosteroid therapy was initiated, resulting in an improvement in both the symptoms and the inflammatory markers. Afterward, the patient received infliximab and underwent surgery to remove ulcerated skin tags following a massive hemorrhagic episode; the subsequent histopathological examination revealed numerous granulomas (Figure 4). After the initiation of infliximab, symptomatic and laboratory improvements were observed.

## DISCUSSION

HPS was first described in 1959, and its incidence is estimated to be between 1 in 500,000 and 1 in 1,000,000.^4^ This complex syndrome is characterized by the following 3 primary manifestations: tyrosinase-positive oculocutaneous albinism, bleeding diathesis due to platelet dysfunction, and systemic complications resulting from the accumulation of ceroid lipofuscin.^5^ Progressive symptoms may include pulmonary fibrosis, renal disease, and enterocolitis.^6^ Schinella et al were the first to describe granulomatous colitis within the context of HPS, documenting the cases of 2 Puerto Rican families affected by HPS.^6^ Among these cases, a total of 5 patients presented with severe colitis. The pathogenesis of bowel involvement in HPS has yet to be fully understood. It has been hypothesized that the development of granulomatous colitis in HPS may be attributed to the accumulation of ceroid-like pigment within intestinal macrophages, resulting in their rupture, leading to the release of lysosomal hydrolases and subsequent tissue damage.^1,3,6–8^ Eleven HPS types have been identified, with type 1 being the most frequent; granulomatous enterocolitis is usually associated with type 1, manifesting in the first and second decades of life and sharing pathological and phenotypic similarities with Crohn's disease.^3,9–11^ In a cohort reported by Hussain et al, among the 24 patients with HPS who underwent endoscopic procedures, 7 (29%) were diagnosed with Crohn's disease, and 2 (8%) also had perianal manifestations.^8^ Other perianal manifestations have also been reported in patients with HPS, including fistulae, abscesses, and skin tags (Table 1).^3,9^ Differential diagnoses of granulomatous colitis also include infectious diseases such as tuberculosis, leishmaniosis, and schistosomiasis, as well as sarcoidosis; sarcoid granulomas are largely devoid of other inflammatory cells and often contain calcified bodies, while paraclinical investigations are necessary to confirm diagnosis, the patient's medical history, along with clinical signs and cues from the morphology of granulomas, can offer valuable clues to the possible etiology.^12^ Colonic tuberculosis is encountered in only 10%–25% of cases; granulomas are typically larger, often confluent, located beneath the ulcerations, and absent in noninflamed mucosa, with detectable caseum in half cases.^13^ Despite the presence of tests such as polymerase chain reaction, interferon-gamma release assay, and biopsies, intestinal tuberculosis can pose a diagnostic challenge and warrants empiric treatment when suspected.^14^ Colonoscopy in HPS may reveal various nonspecific manifestations, such as erosions, friable ulcerations, congestion, pseudopolyps, and hyperemia.^15^ Histopathological observations include granulomas without central necrosis, sometimes with a tendency to merge, and macrophages containing ceroid lipofuscin.^9,15^ Managing HPS-associated colitis can also be challenging; previous reports have shown the inefficacy of antibiotics, mesalazine, and sulfasalazine.^3^ Owing to the clinical similarities between HPS colitis and Crohn's disease colitis, the therapeutic approach is similar.^9^ Azathioprine and 6-mercaptopurine have shown promising results in patients with moderate bowel disease. Surgery also may be necessary in some cases, as Gahl et al reported.^8^ In recent studies, infliximab has been effective in treating granulomatous colitis and perianal fistulas in patients with HPS who did not respond to medical therapies such as antibiotics, corticosteroids, and immunomodulators.^1,11,16,17^ There is also a report of successful outcomes with tacrolimus and vedolizumab in treating HPS-associated colitis.^18^ Although HPS outcome is mainly related to pulmonary fibrosis, its diagnosis relies on high-resolution chest computed tomography in a series of patients with HPS reported by Witkop et al and 4 of 44 (9%) experienced death, which in part attributed to colitis-related complications.^7,19^

**Table 1.** Characteristics of reported patients with granulomatous colitis associated to HPS

Ref.	Yr	Country	*n*	Age in yr	Sex	GI symptoms	Endoscopy findings	HPS	Treatment	Outcome
Alexis et al	2006	United States	4	28	F	Intermittent diarrhea and rectal pain, rectal pain and occasional blood, lower GI bleeding	Colitis with mucopurulent exudate; proctitis	UKN	Infliximab and azathioprine	Improvement
				47	F	Pain and occasional GI bleeding	Proctitis	UKN	Infliximab, 6-MP	Improvement
				33	M	Lower GI bleeding	Moderate rectitis	UKN	Infliximab, 6-MP	Colectomy
				53	M	GI bleeding	Segmental colitis with ulcerations	UKN	Mesalazine, 6-MP, infliximab	Small bowel resection
Felipez et al	2009	United States	1	17	M	Bloody diarrhea	Melanosis, deep colorectal ulcerations	Type 1	6-MP, infliximab	Improvement
Hazzan et al	2006	United States	6	32	F	Chronic diarrhea, lower GI bleeding	UKN	Type 1	UKN	Subtotal colectomy; completion proctectomy + end ileostomy; small bowel resection
				52	M	Lower GI bleeding	UKN	UKN	UKN	Subtotal colectomy + end ileostomy
				30	M	Diarrhea, lower GI bleeding, perianal abscesses	UKN	Type 1	UKN	Subtotal colectomy + ileorectal anastomosis
				4	M	Chronic diarrhea and perianal abscess	UKN	Type 1	UKN	UKN
				29	M	Severe diarrhea	UKN	Type 1	UKN	UKN
				11	M	Chronic diarrhea	UKN	Type 1	UKN	UKN
Kouklakis et al	2007	Greece	1	42	F	Abdominal pain and bloody diarrhea	Segmental colitis with multiple ulcers, deep ileal ulcers	UKN	Azathioprine, infliximab	Improvement
Yoshiyama et al	2009	Japan	1	28	M	UKN	Proctitis	Type 6	Mesalazine, azathioprine, infliximab	Improvement
Mora et al	2011	United States	1	53	M	GI bleeding	UKN	UKN	Mesalazine, 6-MP, infliximab	Improvement

6-MP, 6-Mercaptopurine; F, female; GI, gastrointestinal; M, male; UKN, unknown.

## DISCLOSURES

Author contributions: All of the authors participated in collecting and analyzing the patient's data and designing and writing the report. Hajar Koulali is the article guarantor.

Financial disclosure: None to report.

Informed consent was obtained for this case report.
